# How to Stop Victims’ Suffering? Indirect Effects of an Anti-Bullying Program on Internalizing Symptoms

**DOI:** 10.3390/ijerph16142631

**Published:** 2019-07-23

**Authors:** Benedetta Emanuela Palladino, Annalaura Nocentini, Ersilia Menesini

**Affiliations:** Department of Education, Languages, Intercultures, Literatures and Psychology, University of Florence, Via San Salvi 12, Padiglione 26, 50135 Firenze, Italy

**Keywords:** antibullying intervention, internalizing symptoms, internalizing problems, NoTrap program, cybervictimization, victimization, evidence-based intervention, indirect effects, mediation model, latent growth curve model

## Abstract

Victims of bullying and cyberbullying present internalizing problems, such as anxiety, psychosomatic and depressive symptoms, and are at higher risk of considering or attempting suicide. Researchers have put great effort into developing interventions able to stop bullying and cyberbullying, and thus buffering possible negative consequences. Despite this, only a few of them have investigated the effects of these programs on the psychological suffering of the victims. The NoTrap! program is an Italian evidence-based intervention able to reduce victimization, bullying, cybervictimization and cyberbullying. The aim of the present study is to analyze whether the NoTrap! program can reduce internalizing symptoms through the decrease in both victimization and cybervictimization. Participants were 622 adolescents, enrolled in the 9th grade of eight high schools in Tuscany (experimental group: *N* = 451; control group: *N* = 171). We collected data at three time points: pre-, mid- and post-intervention. Using latent growth curve models, we found that the program significantly predicted the change in internalizing symptoms over time. Furthermore, the mediation model showed that only the indirect effect via cybervictimization was significant. In summary, the program reduced internalizing symptoms within the experimental group successfully, through the decrease in cybervictimization more so than through the mediational effect of decreasing victimization.

## 1. Introduction

Peer victimization is now recognized as a significant risk factor for an individual’s well-being and adjustment. The symptoms that victims display in response to their bullying experience include internalizing symptoms such as depression, anxiety, suicidality, eating disorders and psychosomatic problems [1,2,3,4,5,6]. Similarly, victims in the cyber context show higher levels of anxiety, depression, suicidal ideation, stress, fear, low self-esteem, feelings of anger and frustration, helplessness, nervousness, irritability, somatizations, sleep disorders, suicidal thoughts, and difficulty to concentrate, all of which affect their academic performance and social adjustment [7,8,9,10].

Looking at the consequences, it seems clear that the prevention of bullying and cyberbullying is crucial. Every intervention in this area aims to reduce the short- or long-term impact of these experiences on physical, psychological, and relational planes, and improve the general well-being [4,5,7,8,9,10,11,12,13,14] of the victims.

However, until now, few studies have evaluated whether an antibullying prevention program may also have secondary effects on psychological symptoms and reduce the suffering of the victims [15,16,17,18]. The current study aims to show evidence of the indirect effects of the NoTrap! antibullying program [19] on internalizing symptoms through the decrease of cyber and face-to-face traditional victimization.

### 1.1. Mental Health Consequences of Victimization and Cybervictimization 

In the past three decades, a significant amount of effort has been put forth by researchers analyzing the effects of face-to-face bullying, physically, psychologically, relationally, and in regards to general well-being. As Arseneault and colleagues [1] emphasized in their critical review, bullying is associated, in the short term, with severe symptoms of mental health problems and, furthermore, has long-lasting effects that can persist until late adolescence. That is, bullying appears to contribute to children’s mental health problems on multiple levels.

Recent meta-analyses, largely focused on longitudinal studies, help summarize such findings [4,5,13,14,20,21,22,23,24]. Reijntjes and colleagues took 18 longitudinal studies [5] into account to analyze the role of internalizing problems and their relationship to victimization. The authors concluded that such problems appear to be both antecedents and consequences of peer victimization, constituting a “vicious cycle” that contributes to the elevated stability of peer victimization. Analyzing 37 studies [4], Kim and Leventhal found that any type of major involvement in bullying incidents (as a bully, as a victim or as a bully-victim) increased the risk of suicidal ideation and/or behavior. Bully-victims, victims, and bullies were at significantly higher risk of psychosomatic problems compared to their uninvolved peers [2,11], and victimization was a major childhood risk factor that plays a major role in later depression, even controlling for many other major childhood risks [13]. In their meta-analysis on victimization and suicidal behavior, Holt and colleagues observed a moderate effect size for the significant odds ratio between victimization, and suicidal ideation and behavior. By focusing on the children and adolescents considered as “healthy”, without predispositions for suicidal factors, Katsaras and colleagues [22] found in a meta-analysis that victims of bullying are significantly more likely to present suicidal ideation and attempt suicide, compared to uninvolved participants.

Only a few studies were able to analyze long-term effects of victimization using longitudinal methods that reassessed participants throughout a large portion of their lives. Using data from a longitudinal study of a birth cohort of 1265 individuals who were monitored until their 30th year [25] Gibb and colleagues emphasized that reports of victimization in childhood are associated with higher rates of mental health/adjustment problems (such as major depression, anxiety disorders, suicidal ideation, suicide attempts, alcohol dependence, illicit drug dependence, conduct/antisocial personality disorder, violent offending, property offending, and arrest/court conviction) later in life. Results from the Pittsburgh Youth Study [12], which included a first assessment at 6–7 years and a follow-up at age 19 led the authors to the conclusion that bullying victimization is followed by an increased risk of depression.

In recent years, the important connection between bullying and well-being was further analyzed considering the possible unique, parallel, additional or synergistic effects played by context: what happens when bullying takes place in a cyber-context? The interactions that occur in the virtual world can affect the everyday reality that students experience elsewhere. As for bullying, the growing literature about cyberbullying often highlights the consequences of this phenomenon with regards to the individual’s well-being. Studies [7,8,9,10] have shown that cyber-victims experience anxiety, problematic internet use, suicidal ideation, stress, fear, low self-esteem, feelings of anger and frustration, helplessness, nervousness, irritability, somatization, sleep disturbances, and difficulty concentrating, all of which affect their academic performance. 

Looking at the results of some metanalyses and systematic reviews, it is clear that cyber victimization is significantly and positively related to depression [26] and internalizing problems [27,28], and victims of cyber bullying are significantly more likely to self-harm, present suicidal ideation and attempt suicide [22,29].

### 1.2. The Role of Context 

Research appears to show that the negative consequences of cyberbullying often parallel those of traditional bullying, and a main research question arises: are there unique and particularly troubling aspects of the cyber context that could affect the quality and magnitude of the connection between bullying and its negative consequences? We have to keep in mind that, unlike traditional bullying, the cyber context has specific features that could play a role. Scholars have put great effort into analyzing their impact on bullying, and the consequences that follow [30,31,32]. Cyberbullying can occur at any time, which may heighten children’s perceptions of vulnerability. At the same time, something “hurtful”—e.g., text messages, video, pictures, etc.—can also be distributed quickly to a wide audience and sometimes it is difficult to remove the offensive material definitively.

Although recent research has demonstrated significant connections between involvement in cyberbullying and various psychological health symptoms and difficulties, there is still an open debate as to whether these connections are independent from the involvement in more traditional forms of bullying. The highest risks of poor adjustment were observed in students who were identified as combined bully-victims, in both traditional and cyber contexts [33]. There is a growing body of research investigating the unique effects of traditional and cyber victimization on depression and suicidal ideation among youths. Thus far, the results of these studies are equivocal. Some studies did not find a significant association between cyber victimization and depressive symptoms after checking for the effects of traditional victimization [34,35], while others did [36,37,38]. Bonanno and Hymel [36] found cyber victimization able to explain a small but significant amount of variance in symptoms of depression and suicidal ideation above and beyond that of traditional victimization. Similarly, in a longitudinal study [39] it was found that cybervictimization is an additional risk factor for depressive symptoms over and beyond traditional victimization in adolescents. Unique and multivariate effects were detected in a sample of Italian adolescents [40]: both types of bullying and victimization (traditional and cyber) contributed significantly to explaining externalizing and internalizing symptoms. Fredrick and Demaray [38], in a sample of 9th grade (13- to 16-year-old) adolescents, found that both traditional and cyber victimization were significantly linked to depressive symptoms and the strength of each relation with depressive symptoms was similar. In a recent meta-analysis, Gini and colleagues [28] examined the associations between cyber-victimization and internalizing problems controlling for the occurrence of traditional victimization. They found that they are both uniquely related to internalizing problems and the difference in the relations between each type of victimization and internalizing problems was not statistically significant.

### 1.3. The Effects of Antibullying Interventions on Victims’ Mental Health 

In all the studies cited above, the authors concluded their research by espousing the importance of carrying out effective anti-bullying programs that would have a high benefit/cost ratio in terms of suicide, internalizing symptoms, and so on.

Although there is a growing body of research on intervention programs against bullying and cyberbullying [41,42,43], until now research on secondary effects of antibullying programs is very scarce and controversial [15,16,17,18]. For example, the KiVa program in Finland has a significant impact in reducing students’ anxiety but not their depression, and the change in anxiety was found to be predicted by a reduction of victimization [18]. In the study by Nocentini and colleagues [17], the KiVa antibullying program conducted in Italy showed only a marginally significant decrease in the KiVa group, more so in highly sensitive boys. Also, the study conducted by Juvonen and colleagues [16] supported a moderated effect of the KiVa antibullying program on depression, with stronger effects on the most victimized students. In the Dutch study conducted by Fekkes and colleagues [15], at the end of the first year of their program, as a trend, fewer depressive symptoms were reported in intervention schools than in control schools.

Although it is highly recommended in terms of both awareness about the processes involved and consciousness of the overlap between the two phenomena—and the unique effects they have on the victims’ suffering [28]—none of these studies tested the mediation effects while simultaneously taking both contexts (face to face and online) into account.

### 1.4. The Current Study 

The NoTrap! Program is one of the few interventions that is effectively able to reduce both the act of bullying and its consequent suffering, in both traditional and cyber contexts—i.e., victimization, bullying, cybervictimization and cyberbullying [19,41,42,43,44]. Despite these important results, the effects of victims’ and cybervictims’ suffering is still unclear. The aim of the present study is to analyze the efficacy of the NoTrap! intervention by using a mediational model to study the reduction of internalizing symptoms. More specifically, our goal was to test the effects of indirect intervention on internalizing symptoms via the decrease in victimization and cybervictimization.

## 2. Method 

### 2.1. Participants 

Participants in the study were part of the quasi-experimental trial of the NoTrap! program, conducted during the 2011/2012 school year [19]. The participants were 622 adolescents, enrolled in the 9th grade of 8 high schools throughout Tuscany (provinces of Lucca and Florence); 29.3% of the students attended lyceum high schools, 13.5% attended technical institutes, and 57.2% attended vocational high schools. The majority of students were Italian (85.88%); 6% came from East Europe (mainly Albania and Romania), and the rest were from various other parts of the world; 76% of the sample had passed the previous grade and were attending high school for the first time, while 24% had failed their final exams and were repeating the 9th grade.

The experimental group was composed of 451 adolescents (57% male; mean age = 14.79; standard deviation (SD) = 1.12) attending 22 classes, across 5 high schools. According to the program scheme, 92 students (53.3% male) decided to assume a more involved role in the program by becoming peer educators.

The control group was composed of students who had not received any kind of intervention (*N* = 171; 69% male; mean age = 15.28; SD = 1.15). Three schools accepted to participate as a control group, comprising of a total of 9 classes.

### 2.2. Procedure

Experimental schools were determined through a self-selection inclusion process, and the classes were picked by the school staff. In June 2008, the Province of Lucca and the Ufficio Scolastico Regionale (the regional office of MIUR, Ministero dell’Istruzione, dell’Università e della Ricerca, the Ministry of Education, University and Research) released an invitation to participate in the program, which was sent to all of the high schools in Lucca and Florence (as letters to the schools’ principals). No school accepted to participate with a random selection, so we were unable to conduct a randomized control trial (RCT) design. For this reason, we paired schools that requested to participate as project schools with other control schools, specifically with classes with the same type of curriculum (e.g., we paired vocational schools for mechanics; technical schools for computer sciences, etc.). In September, we asked specific schools to participate as control schools.

No differences were found between experimental and control groups regarding the types of school attended (Lyceum, Technical, Vocational high schools) in our sample (χ²_(2, 622)_ = 1.534; *p* = 0.464), suggesting that the pairing was appropriate.

The steps of the NoTrap! program were the following:

(I) Initial evaluation (questionnaire administration in November 2011).
(II) Teachers training. Specific course on bullying and cyberbullying focusing on what a school can do against bullying and cyberbullying (two meetings in each experimental school, 2 h each); free admission was granted to all teachers of the experimental schools. The goal was to involve schoolteachers and communities, and to start a joint revision (with students) of the school rules and policies on bullying and cyberbullying.
(III) Launch of the project and awareness development. Presentation of the project to the participating classes in order to try to raise awareness and communication on issues related to bullying and cyberbullying (2 h, two combined classes). We used videos and other materials that were developed in the previous editions of the program. The meeting was followed by another meeting with “a special police unit” (i.e., Polizia Postale) psychologist, and focused on the criminal implications of bullying and cyberbullying.
(IV) Selection of peer educators from each participating class through self-nomination.
(V) Day training for peer-educators (8 h) focused on communication skills, social skills in real and virtual interactions, victim and bystander emotions, empathy and coping strategies (e.g., problem-solving on how to deal with bullying and cyberbullying).
(VI) Middle evaluation, after the first adult-led part of the project. Questionnaire administration at the end of February 2012. At this stage peer educators have not yet started the activities: they were only trained by the program staff.
(VII) Face-to-face peer educators activities in their own class (two meetings—2 h each) on: (1) victim and bystander feelings and emotions, and empathy; (2) how to cope in situations of bullying and cyberbullying, from the point of view of the victim and bystander (what can I do if I see an incident of bullying or cyberbullying, or if I am a victim or a cybervictim?). They used problem solving strategies in order to decide a variety of possible solutions and they chose whichever they thought was the best. Small groups were led by peer educators. Each student was given a specific role in order to participate in completing the activity. The groups made posters which were later published on the NoTrap! Facebook page. At the end of the activities the students presented their posters to their classmates, and a discussion was held about the solutions they found and how they felt.
(VIII) Online peer educators’ activities. We created a rotation schedule whereby all online peer educators worked for two weeks as moderators and publishers and Facebook group (called NoTrap!) administrators.
(IX) Final evaluation. The same questionnaire was re-administered at the end of May/start of June 2012 to evaluate the final situation after the peer-led part of the program.
(X) Main conference. Data restitution to the schools and students in October 2012. Before the conference there was a Facebook contest: the class who had gained the most “likes” on the poster they had created during the class activities, won a tablet.

The questionnaires were administered in class by trained researchers during school time (Masters or Ph.D. graduating students). Informed consent procedures consisted of approval by the school, the class council, and the parents: 100% of the families agreed to their children’s participation in the research.

### 2.3. Measures

Victimization: the Florence Bullying/Victimization Scales were used [19]. Each scale consists of 10 items that ask how often respondents have experienced particular behaviors (as perpetrator and victim, separately) in the previous couple of months. For the current study we only used the victimization scale. Each item was evaluated on a 5-point scale ranging from “never” to “several times a week.” The scale was composed of three subscales: physical (4 items; e.g., “I have been beaten up”), verbal (3 items; e.g., “I have been teased”) and indirect (3 items; e.g., “I have been ignored by my schoolmates”) victimization. First and second order Confirmatory Factor Analyses (CFA) showed good fit indices for both scales (see [19]). The overall scale showed good reliability in all three waves of data collection (Pre-intervention: α = 0.76; Middle evaluation: α = 0.80; Post intervention: α = 0.80).

Cybervictimization: the Florence CyberBullying–cyberVictimization Scales (FCBVSs) were used [19,45]. This measure consists of two scales, one for cyberbullying and one for cybervictimization. For the current study we used only the cybervictimization scale, which consists of 14 items that ask how often (in the past couple of months) respondents have experienced a variety of behaviors. Each item was evaluated by a 5-point scale from “never” to “several times a week”. The scale was composed of four subscales: Written-Verbal (4 items; e.g., “I received threatening and insulting text messages”), Visual (3 items; e.g., “I received videos/photos/pictures of embarrassing or personal situations about me on mobile phone”), Impersonation (4 items; e.g., “Someone manipulated my private personal data in order to reuse them”) and Exclusion (3 items; e.g., “I have been excluded from an online group e.g., on chatrooms, Social Network etc.”). First and second order CFA showed good fit indices for both scales (see [45]). The scale showed good reliability in all three waves of data collection (Pre-intervention: α = 0.83; Middle evaluation: α = 0.91; Post intervention: α = 0.92).

Internalizing symptoms: the Youth Self-Report (YSR) [40,46] is a self-report questionnaire for subjects aged between 11–18 years old. The 103 items were evaluated on a three-point scale ranging from 0 (not true) to 2 (very true or often true). The YSR can be scored on various syndrome scales: social withdrawn, somatic complaints, anxiety-depression that together constitute the Internalizing Scale (31 items; e.g., “I cry a lot”; “I lack energy”; “I feel nobody loves me”); delinquent behavior and aggressive behavior together constitute the Externalizing Scale. For the purposes of the present study, we used data about Internalizing Symptoms scale. The scale showed good reliability in all three waves of data collection (Pre-intervention: α = 0.90; Middle evaluation: α = 0.92; Post intervention: α = 0.91).

### 2.4. Overview of Analyses 

All the analyses were conducted via Mplus 7.0 [47] and PASW 18 [48]. All models were evaluated by means of the following overall indices: the chi-square (χ^2^) statistic, the root mean squared error of approximation (RMSEA), and the comparative fit index (CFI). Recommended cut-off points for these measures are 0.08 [49] or 0.06 [50] for RMSEA and 0.90 or 0.95 for CFI [51]. Given the non-normal distribution of data, we applied a logarithmic transformation to all the behavioral variables (victimization and cybervictimization) and used the transformed variables in all subsequent analyses.

The estimation and prediction of longitudinal development of internalizing symptoms were analyzed through latent growth curve modeling. The latent growth factors (slope and intercept) were regressed on the variable program (0 = control group; 1 = experimental group) in order to test if the program can affect the change over time of internalizing symptoms. In order to interpret the differences between control and experimental group in a meaningful way, multiple-group analyses (experimental versus control group) were conducted.

The same steps were conducted in a previous paper about the efficacy of the program [19] on victimization and cybervictimization. We found that the program had a significant effect on both variables. The fit indices of the growth curve models for victimization and cybervictimization showed that all the models fit the data well. The program significantly predicted the slope of victimization and cybervictimization (respectively: β = 0.193, standard error (SE) = 0.06, *p* = 0.003; cybervictimization: β = 0.216, SE = 0.07, *p* = 0.002) but did not significantly predict the intercepts, confirming that the control and the experimental groups exhibited no pre-existing differences (intercept) on these variables. Looking at the multiple group latent growth curves, none of the slope means for the control group were significant (i.e., stability over time), while there was a significant decrease over time (significant negative mean of the slope) in victimization and cybervictimization in the experimental group (for further details about these model results see [19] ). We used the same latent growth curve models of victimization and cybervictimization for the purposes of the present study.

As a subsequent step, we carried out a full mediation process model to test if the change in internalizing symptoms (outcome) is led by the change in victimization and cybervictimization variables (mediators) due to the program (independent variable) [52]. We tested for the indirect effect and if the direct effect (slope internalizing symptoms on program) remained significant when adding the mediators to the model. Specifically, we tested for two paths of indirect effects in the same full model: (1) the program predicts a change in victimization that in turn predicts a change in internalizing symptoms; (2) the program predicts a change in cybervictimization that in turn predicts a change on internalizing symptoms. The paths between the program variable and the three intercepts were tested to be sure of the initial comparability of the two groups in the final model as well. The covariances between each slope and intercept are model paths, along with the covariances between all intercepts and slopes of cybervictimization and victimization.

### 2.5. Ethics 

When the research was planned (in 2011) a formal Ethical Committee was not yet established in our University nor the Italian Association of Psychology was requesting a formal ethical approval. The research was carried out in accordance with the recommendations of the Italian Association of Psychology and we can state that the investigations followed the Declaration of Helsinki principles. The research project was sent and approved by the school and class councils. Parents’ active consent was obtained prior to the questionnaires administration. Parents and students were informed about the confidentiality and anonymity of their responses, that their participation was entirely voluntary, and they were also told that they could withdraw at any time. In Supplementary Materials (Files 1 and 2), the readers can find the letters we sent to the schools and class councils at the beginning of the school year (both control and experimental schools). After their approval, we sent the same information letter to each student’s family and we collected the active consent forms for each participant. We safely stored all these forms at the University of Florence.

## 3. Results 

Descriptive statistics and correlations among the measures are presented in Table 1 and Table 2.

**Figure 1:**
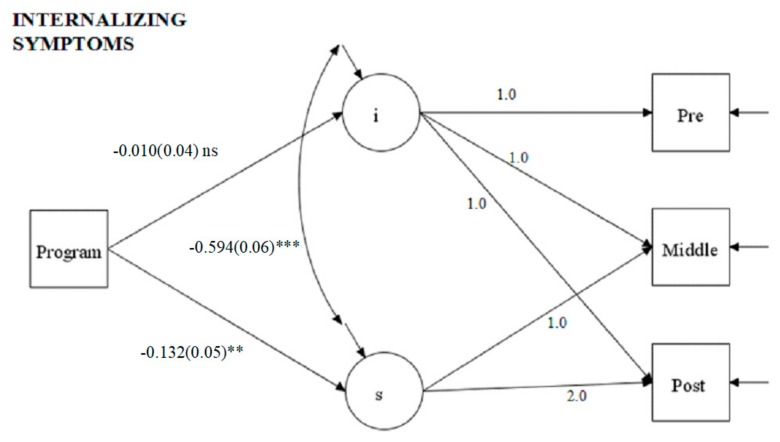
Effects of the program on Internalizing Symptoms growth curves. MODEL FIT: χ² = 0.773; df = 3; *p* = 0.00; comparative fit index (CFI) = 1.000; root mean squared error of approximation (RMSEA) = 0.00. *Note:* The path coefficients and standard errors (in brackets) are standardized esteems (** for *p* < 0.01; *** for *p* < 0.001). *Legend:* “S” stands for internalizing symptoms slope and “I” stands for internalizing symptoms intercept.

Figure 1 presents the fit indices of the model and the effects of the program on the growth curve for internalizing symptoms. The model fit the data well. The program significantly predicts the slope of internalizing symptoms (β = −0.132, SE = 0.05, *p* < 0.001) while it is not a significant predictor of the intercept (β = 0.01, SE = 0.06, *p* = n.s.), confirming that the control and the experimental groups did not significantly differ initially (pre measure – intercept) in this variable either. The covariance between intercept and slope is significant and negative (β = −0.594, SE = 0.06, *p* < 0.001).

Looking at the multiple group latent growth curves, while in the control group the mean of the slope is not significant, in the experimental group there is a significant decrease over time (see Table 3 for the multiple-group estimated components of growth curve and model fit for internalizing symptoms).

**Figure 2:**
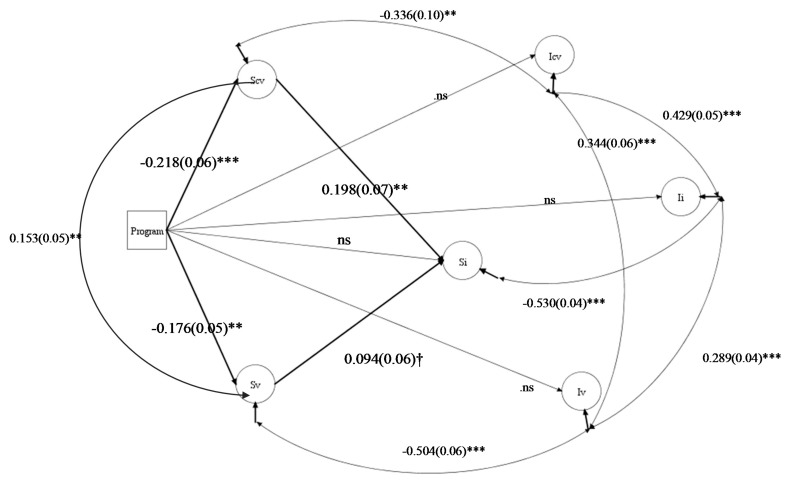
Final full mediational model: direct and indirect effects of the Program on internalizing symptoms. The path coefficients and standard errors (in brackets) are standardized esteems (†for *p* < 0.10; ** for *p* < 0.01; *** for *p* < 0.001). MODEL FIT: χ² = 132.894; df = 29; *p* = 0.00; CFI = 0.918; RMSEA = 0.064. R^2^Si = 7%. TOTAL INDIRECT EFFECT (Program → Si): -0.059(0.020)**. SPECIFIC INDIRECT EFFECTS: VIA Scv = −0.043(0.018)*; VIA Sv = −0.016(0.01) n.s. *Legend:* “Si” stands for internalizing symptoms slope and “Ii” stands for internalizing symptoms intercept; “Sv” stands for victimization slope and “Iv” stands for victimization intercept; “Scv” stands for cybervictimization slope and “Icv” stands for cybervictimization intercept. *Note:* For the readability of the figure only the paths between latent variables and the independent variable are shown.

In Figure 2, we reported fit indices of the final mediation process model and the estimates of all the paths between the latent variables (slopes and intercepts), and between them and the independent variable (program). All the covariances between each slope and intercept are significant and negative. The initial levels of the three latent growth curves exhibit significant positive relationships: internalizing symptoms intercept strongly covaries both with cybervictimization intercept (β = 0.429, SE = 0.051, *p* < 0.001) and victimization intercept (β = 0.289, SE = 0.037, *p* < 0.001). Significant covariations both for intercepts (β = 0.344, SE = 0.056, *p* < 0.001) and slopes (β = 0.153, SE = 0.047, *p* < 0 0.01) between victimization and cybervictimization were found. No effects of the program were found on the three intercepts, confirming the comparability of the experimental and control groups at the pre-measure, in the final model.

The program significantly predicts both slopes of victimization (β = –0.176, SE = 0.052, *p* < 0.01) and cybervictimization (β = –0.218, SE = 0.059, *p* < 0.001), confirming the efficacy of this program in reducing both, in the final mediation process model. On the contrary, when introducing the latent growth curves of those variables in the model, the direct effect to internalizing symptoms is no longer significant (β = –0.009, SE = 0.10, *p*= n.s.). The slope of cybervictimization is a significant predictor of the slope of internalizing symptoms (β = 0.195, SE = 0.065, *p* < 0.01) while for the victimization slope, we found a marginally significant effect (β = 0.094, SE = 0.056, *p* < 0.10).

We tested for the indirect effect (program variable on internalizing symptoms slope through cybervictimization and victimization slopes). Overall, the total indirect effect is significant (β = –0.59, SE = 0.02, *p* < 0.01). Upon analyzing the two paths specifically, the only significant indirect effect which led to a decrease in internalizing symptoms appears to be the decrease in cybervictimization led by the program (β = –0.043, SE = 0.018, *p* < 0.01). For victimization the indirect effect is not significant (β = –0.016, SE = 0.011, *p* = 0.13) (for the readability of the paper, the results of the two separate mediation models (cybervictimization and victimization) were not included. However, the indirect paths from the program to the slope of internalizing symptoms were significant both for cybervictimization (β = –0.035, SE = 0.016, *p* < 0.05) and victimization (β = –0.024, SE = 0.012, *p* < 0.05)).

Summarizing, the decrease in cybervictimization is a significant mediator of the efficacy of the program in reducing internalizing symptoms over and above the effects (decrease) in victimization.

## 4. Discussion 

Starting from the increased demand for an evidence-based framework that can inform interventions and policies against bullying and cyberbullying [53,54], the aim of the present study was to analyze the efficacy of the NoTrap! program in terms of effects on internalizing symptoms. The general goals of interventions against bullying and cyberbullying is to impact youths’ physical, psychological, relational and general well-being [4,5,7,8,9,10,11,12,13,14]. Researchers and professionals have put great effort towards developing interventions to stop bullying and cyberbullying, and thus buffering the possible negative consequences. Despite this, most studies limit themselves to analyzing the effects of reducing bullying and cyberbullying. Only a few of them have investigated the effects of the programs on the psychological suffering of the victims [15,16,17,18].

On the basis of two recent metanalyses [41,43] the NoTrap! program [19] is one of the few interventions available in the scientific literature able to reduce significantly both bullying and victimization in the scholastic as well as the cyber context. In order to improve our understanding of the efficacy of this program, in the present paper we tested for the effects on internalizing symptoms: we found that it was efficacious in reducing the level of suffering (i.e., internalizing symptoms) in the experimental group, while we found a general stability in the control group. Next, we tested both direct and indirect effects in a full mediation model, to understand if this decrease in internalizing symptoms was due to the program’s effectiveness in reducing victimization and cybervictimization. We found that the decrease in victimization and cybervictimization completely accounted for the effect of the program on internalizing symptoms: only an indirect effect remained significant while the direct one was no longer. Looking in depth at the significant indirect effect, we found that only the path through the cybervictimization slope was significant. In other words, the program was efficacious in reducing internalizing symptoms in the experimental group through the decrease in cybervictimization over and above the effect of the decrease in victimization.

Aside from the evidence-based framework [55,56], these results can be also discussed within the debate over the overlap between the bullying and cyberbullying constructs and their relation with internalizing symptoms [28,57,58,59,60,61]. Literature showed that beside the overlap between the two phenomena, both contexts of victimization are uniquely related to internalizing problems and, therefore, both offer a unique understanding to the link between peer victimization and internalizing problems [28,36,40]. It is quite the same in our data: in the final model the three intercepts in our model are strictly correlated, although the stronger relation is between cybervictimization and internalizing symptoms intercepts. Additionally, we found that the decrease in cybervictimization led to a decrease to internalizing symptoms while the reduction in victimization was not significantly related to the decrease in internalizing symptoms. These findings, and specifically the absence of the last mentioned path, could be discussed by looking at the age of our sample (i.e., adolescents). Specifically, in the aforementioned meta-analyses of the associations between cybervictimization and internalizing problems, Gini and colleagues [28] found a moderation linked to age: the link between cybervictimization and internalizing problems became stronger with age. Therefore, it could be that the heightened effect of cybervictimization on internalizing symptoms is due to the age group of the participants in this study. Additionally, it should be noted that the participants to the intervention were attending their first year of high school, thus were just starting to form relationships with their new classmates when the intervention started.

As an important practical issue, we want to underline the evident importance of taking cyber context into consideration, not simply absorbing cyberbullying under the traditional construct of bullying [58,59,62]. Given the substantial overlap between traditional and cyber forms of victimization, and the results on the impact that reducing cybervictimization has on internalizing symptoms, the importance of prevention approaches which address both types seems confirmed. In fact, our results suggested that maintaining dual focus on both contexts appears to be more adequate. The NoTrap! program is focused on both contexts of bullying and, looking at the overall results we found, we can confirm how this approach may be promising in terms of impacting the victims’ suffering as well.

### Limitations and Future Studies

Before arriving to the conclusion, it is important that we acknowledge some limitations of this research. First, we lacked an important aspect of evidence-based evaluation: the experimental and control samples were only paired based on the schools’ characteristics (i.e., matched control design). In other words, although we tried, the assignment to one or another condition was not randomized (see the procedure section). Related to this issue, another possible bias can be due to the self-selection inclusion process of experimental schools. For instance, it is possible that a higher risk population missed the opportunity to be involved in the intervention. For this reason, in the analyses, we tested for differences in the two groups and controlled for the possible effects of the sampling (i.e., effects of the program on the intercepts in the growth curves model as well in the final one). Although the methods we used can be considered at least acceptable, it would be desirable to replicate our findings using RCTs.

Second, data have been collected in 2011/2012 and consequently we should consider possible changes in the nature of online risky behavior. It would be desirable to replicate our findings with new experimental trials especially when we consider how fast Information and Communication Technologies (ICTs) and the online context evolve nowadays. This future plan is also relevant in order to verify whether the mechanisms described in the study remain the same across different virtual contexts.

Lastly, because of the complexity of the models used to test the effects of the program on internalizing symptoms, no gender analyses were performed. Gender differences are often present when consulting the literature on the effects of interventions on internalizing problems [63]. Although the program appears to apply similarly to both genders (see [19]), further analyses on the effect it has on genders should be conducted to disentangle the possible biases in our results on internalizing symptoms.

## 5. Conclusions

Despite its limitations, this study integrates previous knowledge and gives some relevant suggestions to researchers and practitioners currently working on the prevention of bullying and cyberbullying. Using the standards of evidence [55], we gave special attention to a neglected aspect of the NoTrap! program: the impact on the victims’ suffering. The results we obtained allow us to say that it was efficacious in counteracting the bullying and cyberbullying phenomena and, in turn, buffered internalizing symptoms, thanks to its impact on cybervictimization, over and above traditional victimization.

## Figures and Tables

**Table 1:** Descriptive statistics (means, standard deviation (SD) and *N* size) in the three waves of data collection for victimization, cybervictimization, and internalizing symptoms.

Variable	Group	Pre Measure			Middle Measure			Post Measure		
		Mean	SD	*N*	Mean	SD	*N*	Mean	SD	*N*
**Victimization**	Experimental	0.109	0.114	389	0.091	0.110	372	0.059	0.086	338
	Control	0.093	0.098	130	0.106	0.126	141	0.090	0.121	112
**Cybervictimization**	Experimental	0.044	0.079	378	0.039	0.092	363	0.015	0.041	323
	Control	0.041	0.068	129	0.043	0.099	141	0.043	0.111	108
**Internalizing Symptoms**	Experimental	11.86	9.38	373	11.24	9.68	345	10.03	8.37	312
	Control	12.59	8.87	125	12.21	10.59	136	11.82	11.82	108

**Table 2:** Correlations between victimization, cybervictimization and internalizing symptoms.

Variable	Victimization			Cybervictimization			Internalizing Symptoms		
	1. Pre	2. Middle	3. Post	4. Pre	5. Middle	6. Post	7. Pre	8. Middle	9. Post
**1.**	1	0.606	0.322	0.173	0.113	0.043	0.086	0.143	0.175
**2.**	0.433	1	0.604	0.227	0.133	0.186	0.219	0.237	0.149
**3.**	0.374	0.485	1	0.319	0.504	0.486	0.342	0.341	0.326
**4.**	0.378	0.351	0.254	1	0.379	0.425	0.335	0.247	0.147
**5.**	0.156	0.385	0.232	0.330	1	0.649	0.399	0.254	0.271
**6.**	0.116	0.151	0.207	0.373	0.464	1	0.338	0.329	0.449
**7.**	0.393	0.234	0.228	0.459	0.303	0.151	1	0.772	0.628
**8.**	0.312	0.295	0.242	0.408	0.482	0.308	0.685	1	0.651
**9.**	0.086	0.212	0.168	0.192	0.279	0.224	0.376	0.548	1

Note: Data for control group appears above the diagonal and data for experimental group appears below the diagonal.

**Table 3:** Multiple-group estimated components (unstandardized) of growth curves and models’ fit for victimization, cybervictimization and internalizing symptoms.

Group	Mean Slope	Var. Slope	Mean Intercept	Var. Intercept	Covar. (Int and Slope)	χ² (Each Group)	χ²	Df ^#^	*p*	CFI	RMSEA (90 perc. C.I.) Probability ≤ 0.05
***INTERNALIZING SYMPTOMS***											
**CONTROL**	−0.254 (0.425) ns	12.142 (4.84) *	12.204 (0.742) ***	78.26 (8.62) ***	−9.407 (4.78) *	0.022	0.841	4	0.93	1.000	0 (0–0.24) 0.98
**EXP.**	−0.958 (0.265) ***	20.72 (4.19) ***	11.954 (0.478) ***	89.26 (8.26) ***	−28.83 (5.06) ***	0.819					

*Note*: For all the variables N size is: Control Group = 165; Experimental Group = 433. ^#^ Differences in degree of freedom in models are due to fixed parameters for improving the fit of models (* for *p* < 0.05; *** for *p* < 0.001).

## Supplementary Materials

The following are available online at https://www.mdpi.com/1660-4601/16/14/2631/s1: File 1: Letter for experimental schools, File 2: Letter for control schools.
