# Supplementary material for: How to Stop Victims’ Suffering? Indirect Effects of an Anti-Bullying Program on Internalizing Symptoms
Source: Int J Environ Res Public Health. 2019 Jul 23;16(14):2631. doi: 10.3390/ijerph16142631 (PMC6678412; doi:10.3390/ijerph16142631)
Supplement: Supplementary file 1 [file ijerph-16-02631-s001.zip › 552361-sumplementary/Letter - control schools.pdf]

DOCUMENT 2

Università degli Studi di Firenze

Dipartimento di Psicologia

Firenze, 17/11/2011

Alla cortese attenzione del Dirigente e del collegio Docenti
della scuola XXXXXXXXXXXXXXXX

Gentile preside e gentili docenti

Il progetto di intervento *"Noncadiamointrappola: per non finire nella rete! Sostegno e collaborazione tra pari nel mondo reale e online"* promosso dalla Provincia di Lucca, servizio Politiche Sociali e dal Laboratorio di Studi Longitudinali del Dipartimento di Psicologia dell'Università di Firenze a cui la vostra scuola partecipa in qualità di scuola di controllo, nasce con l'obiettivo di ridurre i comportamenti di bullismo e cyberbullismo.

In vista di una validazione del presente modello di intervento, sarà valutata la presenza di tali fenomeni, l'andamento nel tempo e l'efficacia del progetto stesso nel ridurre la presenza di tali comportamenti attraverso dei questionari somministrati alla classe. Le rilevazioni tramite questionari saranno a cura di ricercatori, tirocinanti e studenti laureandi della Facoltà di Psicologia dell'Università di Firenze e dovrebbero avvenire in tre momenti: nel periodo fine novembre-dicembre, a febbraio e a maggio-giugno.

In particolare, saranno indagati diversi aspetti relativi ai comportamenti e agli atteggiamenti connessi al bullismo e al cyberbullismo, la qualità delle relazioni tra i ragazzi a scuola, l'adattamento scolastico, le coping strategies, l'empatia e il supporto sociale percepito.

Per le famiglie e per gli insegnanti, la sottoscritta, responsabile del progetto, garantisce il pieno rispetto della privacy dei ragazzi, i cui dati verranno trattati solo a livello di gruppo ed inseriti attraverso i codici che saranno loro assegnati ai sensi della Legge n.675 del 31.12.1996 e del D.L. 196/2003.

50135 FIRENZE – Via San Salvi, 12 – Padiglione 26 - Tel. 055 6237811 – Fax 055 6236047
e-mail:psicosegr@psico.unifi.it - Partita Iva 01279680480

Università degli Studi di Firenze

Dipartimento di Psicologia

Data la minore età degli studenti, **è necessaria l'autorizzazione dei genitori** a partecipare al progetto. Vi chiediamo pertanto di chiedere tali autorizzazioni (in allegato) il prima possibile in modo che durante la prima rilevazione siano disponibili.

Ringraziando sentitamente per la collaborazione, rimaniamo a disposizione per eventuali necessità.

Cordiali saluti

La responsabile del progetto di ricerca
Prof.ssa Ersilia Menesini

L'équipe del progetto
Dott.ssa Benedetta E. Palladino
Dott.ssa Annalaura Nocentini

50135 FIRENZE – Via San Salvi, 12 – Padiglione 26 - Tel. 055 6237811 – Fax 055 6236047
e-mail:psicosegr@psico.unifi.it - Partita Iva 01279680480
