# Supplementary material for: How to Stop Victims’ Suffering? Indirect Effects of an Anti-Bullying Program on Internalizing Symptoms
Source: Int J Environ Res Public Health. 2019 Jul 23;16(14):2631. doi: 10.3390/ijerph16142631 (PMC6678412; doi:10.3390/ijerph16142631)
Supplement: Supplementary file 1 [file ijerph-16-02631-s001.zip › 552361-sumplementary/Letter - experimental schools.pdf]

Document 1

Università degli Studi di Firenze

Dipartimento di Psicologia

Firenze, 17/11/2011

Alla cortese attenzione del Dirigente e del collegio Docenti
della scuola [redacted] ....

Gentile preside e gentili docenti

Il progetto di intervento *"Noncadiamointrappola: per non finire nella rete! Sostegno e collaborazione tra pari nel mondo reale e online"* promosso dalla Provincia di Lucca, servizio Politiche Sociali e dal Laboratorio di Studi Longitudinali del Dipartimento di Psicologia dell'Università di Firenze a cui la vostra scuola partecipa, nasce con l'obiettivo di ridurre i comportamenti di bullismo e cyberbullismo. A partire da un momento iniziale di sensibilizzazione, rivolto a tutta la classe, il progetto si articolerà in diverse fasi durante tutto l'anno scolastico (vedere documento allegato). In particolare, alcuni studenti in ogni classe assumeranno il ruolo di peer educator e, dopo un training specifico, lavoreranno con i propri compagni sia sul versante delle emozioni sia quello delle possibili risposte da mettere in atto per prevenire e contrastare i comportamenti aggressivi tra pari.
In vista di una validazione del presente modello di intervento, sarà inoltre valutata l'efficacia del progetto stesso nel ridurre la presenza di comportamenti legati al bullismo e al cyberbullismo attraverso dei questionari somministrati alla classe. In particolare, saranno indagati diversi aspetti relativi ai comportamenti e agli atteggiamenti connessi a tali fenomeni, la qualità delle relazioni tra i ragazzi a scuola, l'adattamento scolastico, le coping strategies e l'empatia.

Per le famiglie e per gli insegnanti, la sottoscritta, responsabile del progetto, garantisce il pieno rispetto della privacy dei ragazzi, i cui dati verranno trattati solo a livello di gruppo ed inseriti attraverso i codici che saranno loro assegnati ai sensi della Legge n.675 del 31.12.1996 e del D.L. 196/2003.

50135 FIRENZE – Via San Salvi, 12 – Padiglione 26 - Tel. 055 6237811 – Fax 055 6236047
e-mail:psicosegr@psico.unifi.it - Partita Iva 01279680480

Università degli Studi di Firenze

Dipartimento di Psicologia

Data la minore età degli studenti, **è necessaria l'autorizzazione dei genitori** a partecipare al progetto, come da schema riportato in allegato. Vi chiediamo pertanto di chiedere tali autorizzazioni il prima possibile in modo che durante la prima rilevazione siano disponibili.

Le rilevazioni tramite questionari saranno a cura di ricercatori, tirocinanti e studenti laureandi della Facoltà di Psicologia dell'Università di Firenze e dovrebbero avvenire in tre momenti: nel periodo fine novembre-dicembre, a febbraio e a maggio-giugno.

Ringraziando sentitamente per la collaborazione, rimaniamo a disposizione per eventuali necessità.

Cordiali saluti

La responsabile del progetto di ricerca
Prof.ssa Ersilia Menesini

L'équipe del progetto
Dott.ssa Benedetta E. Palladino
Dott.ssa Annalaura Nocentini

50135 FIRENZE – Via San Salvi, 12 – Padiglione 26 - Tel. 055 6237811 – Fax 055 6236047
e-mail:psicosegr@psico.unifi.it - Partita Iva 01279680480
